# Supplementary material for: Modeling the kinetics of the neutralizing antibody response against SARS-CoV-2 variants after several administrations of Bnt162b2
Source: PLoS Comput Biol. 2023 Aug 7;19(8):e1011282. doi: 10.1371/journal.pcbi.1011282 (PMC10434962; doi:10.1371/journal.pcbi.1011282)
Supplement: S1 Appendix — (PDF) [file pcbi.1011282.s004.pdf]

Appendixes for ”Modeling the kinetics of the neutralizing antibody response against SARS-CoV-2 variants after several administrations of Bnt162b2”

Quentin Clairon[1,2,3◕¤], Mélanie Prague[1,2,3◕¤], Delphine Planas[3,4◕], Timothée Bruel[3,4 ‡], Laurent Hocqueloux[5‡], Thierry Prazuck[5‡], Olivier Schwartz[3,4 ‡], Rodolphe Thiébaut[1,2,3 ‡*], Jérémie Guedj[6 ◕]

1 Université de Bordeaux, Inria Bordeaux Sud-Ouest, Bordeaux, France
2 Inserm, Bordeaux Population Health Research Center, SISTM Team, UMR1219, Bordeaux, France
3 Vaccine Research Institute, Créteil, France
4 Virus and Immunity Unit, Institut Pasteur, Université de Paris Cité, CNRS UMR3569, Paris, France
5 Service des Maladies Infectieuses et Tropicales, Centre Hospitalier Régional, Orléans, France
6 Université Paris Cité, IAME, Inserm, Paris, France

◕These authors contributed equally to this work.
‡These authors also contributed equally to this work.
¤Current Address: Bordeaux Population Health Research Center, Université de Bordeaux, 146 rue Léo Saignat,33076 Bordeaux cedex, France.
* rodolphe.thiebaut@u-bordeaux.fr

Appendix A: simplified mechanistic model derivation

Appendix A.1: simplification procedure

As mentioned in Section Materials and Methods, the original ODE:

$$\begin{aligned}
\dot{V} &= -\delta_V V \\
\dot{M} &= \rho V - \mu V M \\
\dot{\widetilde{S}} &= \mu V M - \delta_S \widetilde{S} \\
\dot{Ab} &= \theta \widetilde{S} - \delta_{Ab} Ab
\end{aligned} \tag{1}$$

is not structurally identifiable in our partially observed framework. As observed in Balelli et al. [1], M settles to an equilibrium $\overline{M_k}$ after $k-$th injection, so we replaced M by $\overline{M_k}$ on $[\mathbf{t_k}, \mathbf{t_{k+1}}]$ to get the simplified model:

$$\begin{aligned}
\dot{\widetilde{S}} &= \mu V_0 \overline{M_1} G(t) - \delta_S \widetilde{S} \\
\dot{Ab} &= \theta \widetilde{S} - \delta_{Ab} Ab
\end{aligned} \tag{2}$$

where we have also replaced V by its closed form $V(t) = V_0 e^{-\delta_V (t-\mathbf{t_k})}$ and where $G(t) := f_{\overline{M_k}} e^{-\delta_V (t-\mathbf{t_k})}$ on interval $[\mathbf{t_k}, \mathbf{t_{k+1}}]$ and $f_{\overline{M_k}} := \frac{\overline{M_k}}{\overline{M_1}}$. Despite reducing the number of unobserved state-variables and unknown parameters, $\mu V_0 \overline{M_1}$ and θ in the ODE (2) are not jointly structurally identifiables. Indeed, by following the lines of [2]

giving necessary conditions for ODE structural identifiability, we derive that every ODEs:

$$\begin{aligned}\dot{S_a} &= a\left(\mu V_0 \overline{M_1}\right) G(t) - \delta_S S_a \\ \dot{Ab} &= \tfrac{\theta}{a} S_a - \delta_{Ab} Ab\end{aligned}$$

ruling the behavior of $S_a := a\widetilde{S}$ would predict the same evolution for Ab, no matter the value of a. To remove this problem, we consider the ODE :

$$\begin{aligned}\dot{S} &= G(t) - \delta_S S \\ \dot{Ab} &= \vartheta S - \delta_{Ab} Ab\end{aligned} \tag{3}$$

corresponding to $a = \left(\mu V_0 \overline{M_1}\right)^{-1}$ and ruling the rescaled variable $S := \left(\mu V_0 \overline{M_1}\right)^{-1} \widetilde{S}$ in which the parameter $\vartheta := \mu V_0 \overline{M_1} \theta$ merges the previous non-identifiable parameters into one, thus re-establishing identifiability.

Regarding ϑ interpretation, let us remind that first injection is assumed to be the first antigen encounter for vaccinated subjects, so $S(\mathbf{t_1}) = Ab(\mathbf{t_1}) = 0$. From this, let us notice that $\ddot{Ab}(\mathbf{t_1}) = \vartheta \dot{S}(\mathbf{t_1}) - \delta_{Ab} \dot{Ab}(\mathbf{t_1}) = \vartheta\left(G(\mathbf{t_1}) - \delta_S S(\mathbf{t_1})\right) - \delta_{Ab}\left(\vartheta S(\mathbf{t_1}) - \delta_{Ab} Ab(\mathbf{t_1})\right) = \vartheta$, so ϑ is the initial acceleration in antibody production after first antigen encounter.

Appendix A.2: competing models

The chosen mechanistic model was build on the steady state approximation $M(t) \simeq \overline{M_i}$ to avoid both practical and structural identifiability issues. This model was chosen for statistical and dynamical reasons over more complex ones we expose here.

Even without this approximation, the original ODE have the following structurally identifiable reformulation on each interval $[\mathbf{t_k}, \mathbf{t_{k+1}}]$:

$$\begin{cases}\dot{\overline{M}} = f_{\rho_k} e^{-\delta_V (t-\mathbf{t_k})} - \mu_S e^{-\delta_V (t-\mathbf{t_k})} \overline{M} \\ \dot{\overline{S}} = \mu_S e^{-\delta_V (t-\mathbf{t_k})} \overline{M} - \delta_S \overline{S} \\ \dot{Ab} = \theta \rho_1 \overline{S} - \delta_{Ab} Ab\end{cases} \tag{4}$$

with $\overline{M} = \rho_1^{-1} M$ and $\overline{S} = \rho_1^{-1} S$ if we assume the generation rate ρ is piecewise constant and given by $\rho = \rho_1 f_{\rho_k}$ between $[\mathbf{t_k}, \mathbf{t_{k+1}}]$. At the contrary to the simplified model (3), the ODE (4) still assumes that M undergoes a transient phase which may slow down Ab production right after each injection. To further explore the effect of delay between antigen injection and the start of antibody production, and its consistency with our available data, we also consider the model:

$$\begin{cases}\dot{M} = \rho e^{-\delta_V (t-\mathbf{t_k})} - \alpha M \\ \dot{N} = \alpha M - \mu_S A N \\ \dot{S} = \mu_S e^{-\delta_V (t-\mathbf{t_k})} N - \delta_S S \\ \dot{Ab} = \theta - \delta_{Ab} Ab\end{cases}$$

in its structurally identifiable formulation:

$$\begin{cases}\dot{\overline{M}} = f_{\rho_k} e^{-\delta_V (t-\mathbf{t_k})} - \alpha \overline{M} \\ \dot{\overline{N}} = \alpha \overline{M} - \mu_S A \overline{N} \\ \dot{\overline{S}} = \mu_S e^{-\delta_V (t-\mathbf{t_k})} \overline{N} - \delta_S \overline{S} \\ \dot{Ab} = \theta \rho_1 \overline{S} - \delta_{Ab} Ab\end{cases} \tag{5}$$

with $\overline{M} = \rho_1^{-1} M$, $\overline{N} = \rho_1^{-1} N$ and $\overline{S} = \rho_1^{-1} S$. In ODE (5), the compartment $\overline{N}$ represents the latency, quantified by the parameter α, between the generation of the memory compartment and its ability to differentiate into secreting cells.

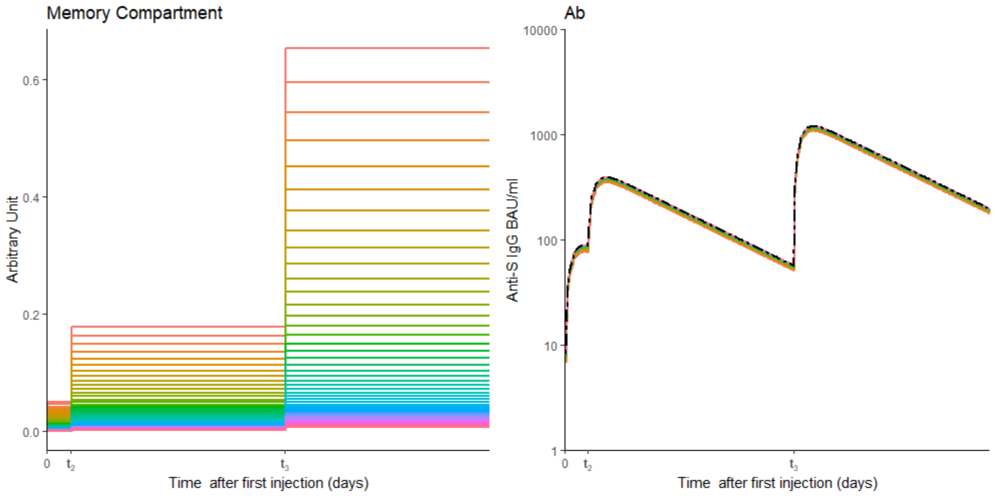

Fig 1. Reconstituted trajectories with ODE (4) for μ_S value ranging from 20 to 200. Left: Memory Compartment M. Right: Antibody concentration, the dashed black line represent the estimated prediction from ODE (3).

We estimate the parameters of ODEs (3)-(4)-(5) to quantify if our available data drives us to statistically account for the existence of additional delay between antigen injection and antibody production comparing to ones already induced by the simplest model (3). The estimation was made from antibody concentration data only to avoid over-fitting phenomena in which the more complex ODEs compensate the error of the neutralization model. Comparing to ODE (3), the models (4)-(5) do not contain $\left(f_{\overline{M_2}}, f_{\overline{M_3}}\right)$ but involve respectively the additional parameters $(f_{\rho_2}, f_{\rho_3}, \mu_S)$ and $(f_{\rho_2}, f_{\rho_3}, \mu_S, \alpha)$. As in the main paper, we profile on (δ_V, δ_S). Not only we face difficulty to estimate μ_S but the simplest ODE (3) gives us the lowest AIC=16.5, followed by ODE (5) with AIC=17.6 and finally AIC=18.3 for ODE (4).

To illustrate why such delay does not seem relevant on a dynamical point of view and why M steady state assumption is a reasonable approximation, we reconstruct M and Ab evolution predicted by estimated model (4). Moreover, we compare these predictions with the ones given by the simplified ODE (3) for parameter values consistent with the estimation of model (4). Let us note that estimation of μ_S in ODE (4) only gives us the upper bound $\mu_S > 20$ because of the already mentioned practical identifiability issues. That is why we plot in Figure 1-left, M prediction corresponding to different values of μ_S ranging from 20 to 200 to account for the diversity of possible trajectories. Using the steady state approximation $\overline{M_k} = \frac{f_{\rho_k}}{\mu_S}$ we derive the relationships $f_{\overline{M_k}} := \frac{f_{\rho_k}}{\mu_S} \times \frac{\mu_S}{f_{\rho_1}} = f_{\rho_k}$ and $\vartheta = \theta\rho_1\mu_S\frac{f_{\rho_1}}{\mu_S} = \theta\rho_1$. Thus, we can use the estimates $(\widehat{\delta_{Ab}}, \widehat{\delta_S}, \widehat{\delta_V}, \widehat{\theta\rho_1}, \widehat{f_{\rho_k}})$ obtained from ODE (4) as surrogate values in ODE (3) for parameters $(\widehat{\delta_{Ab}}, \widehat{\delta_S}, \widehat{\delta_V}, \widehat{\vartheta}, \widehat{f_{\overline{M_k}}})$ to generate Ab trajectory. We plot in Figure 1-right the predicted Ab trajectories for μ_S ranging from 20 to 200 and the only corresponding prediction from ODE (3). For any μ_S value, M quickly settles to its steady-state and the corresponding Ab prediction does not differs from the ones predicted by the simpler ODE (3), supporting its role as relevant practically identifiable approximation of (4).

Appendix B: tested models for neutralization activity

Before settling to the chosen model for neutralization, different expressions for $F(\nu, t)$ have been tested corresponding to different hypotheses on neutralization kinetic. Our goal is to unveil key factors acting on the dynamic of observed gain in neutralization with respect to time. For this, we compare two hypotheses:

H1 no gain from sequential injection, the main factor is the elapsed time since first antigen encounters,

$\widetilde{\mathbf{H}}$ injection-dependent gain, the main factor is the number of exposition to antigen.

Under $\widetilde{\mathbf{H}}$ more precise scenario can be investigated. In particular, does some VoCs benefit more from this repeated injections than others? After the first dose there are differences between neutralization level between VoCs, but additional injections can increase Memory B-cells repertoire diversity [3, 4]. Thus, for VoCs with major mutations comparing to the strain toward which the vaccine was primarily directed, an higher relative gain from a new dose can be expected if new B-cells populations emerge. To account for this, we divide $\widetilde{\mathbf{H}}$ into two-sub hypotheses:

H2 injection-dependent BUT variant-independent gain,

H3 injection-dependent AND variant-dependent gain.

The function $F(\nu, t)$ will change according to the tested hypothesis:

H1 $F(\nu, t) = \gamma f_\nu \times \frac{t}{\beta_\nu + t}$,

H2 $F(\nu, t) = \gamma f_\nu (\mathbb{1}_{t<\mathbf{t_2}} + f_2 \mathbb{1}_{t\in[\mathbf{t_2};\mathbf{t_3}]} + f_3 \mathbb{1}_{t\geq \mathbf{t_3}})$,

H3 $F(\nu, t) = \gamma f_\nu (\mathbb{1}_{t<\mathbf{t_2}} + f_2 g_{2,\nu} \mathbb{1}_{t\in[\mathbf{t_2};\mathbf{t_3}]} + f_3 g_{3,\nu} \mathbb{1}_{t\geq \mathbf{t_3}})$.

For **H1,** $\gamma f_\nu = \lim_{t \longmapsto +\infty} F(\nu, t)$ represents the maximal neutralization capacity per antibody concentration unit for each VoCs. Parameter β_ν is the required time to move from 0 to $\frac{\gamma f_\nu}{2}$. It quantifies the speediness of gain, the smaller β_ν the quicker the gain will be. **H2** is a simplified version of **H3** corresponding to $g_{2,\nu} := 1$ and $g_{3,\nu} := 1$ that is a constant relative gain for all VoCs.

Before estimation, we made simplifying assumptions on some parameter values for practical identifiability purposes. For **H1**, we assume β_ν is common to all variants i.e. $\beta_\nu := \beta$. For **H3**, we were unable to estimate $g_{2,\nu}$ because most of neutralization data for Omicron were left-censored before third injection, so we restrict testing to $g_{2,\nu} := 1$ thus retrieving the retained model in the main paper with $g_{3,\nu} := g_\nu$. We then proceed to parameter estimation for each neutralization model corresponding to hypotheses **H1- H2- H3** similarly as described in Section Methods with the same underlying mechanistic model (3) for Ab evolution. These estimation leads to significantly different Akaike Information criteria (AIC) values. For each **H1**, we end up with AIC=1786, for **H2** AIC=1747 and for **H3** AIC=1627. This drives us to choose **H3** over **H1- H2** that is the repeated injections as the main driver of affinity gain.

Finally, our assumption about the linear relationship between ED_{50}^{ν} and Ab can be seen as too simple. In particular, it may miss saturating effect when antibody concentration is important and competition for free viruses can emerge. To account for this, we test the sigmoïd model

H4 $ED_{50}^{\nu} = F(\nu, t) \frac{Ab^n}{Ab^n + \theta_\nu^n}$ with $F(\nu, t)$ retained from **H3.**

Its estimation leads to AIC=1691 that is, higher than **H3**. Thus, we choose the simpler linear model which still constitutes a relevant approximation of a sigmoïd outside the range of low and high values.

	Second Dose		Third Dose	
	HCWs N=89	OPs N=62	HCWs N=81	OPs N=56
Men	35%	57%	39%	68%
Age	41 (35–50)	79 (73–86)	41 (35-51)	78 (73-83)
Second injection time (days)	97 (91–103)	78 (45–86)	97 (91–102)	76 (45-85)
Third injection time (days)	-	-	210 (200-241)	169 (160-231)
BNT162b2 first/second dose	99%	87%	98%	82%
BNT162b2 third dose	-	-	52%	40%

Table 1. Summary of measured patients after second and third injection. Results are given in percentage or in the format: Median (Interquartile range). The informations are taken from Table 1 in Brockman et al. [5] and Table 1 in Mwimanzi et al. [7] where they originally appear.

Appendix C: internal model assessment

One way to check the model validity in an internal way (i.e using only the training data set) is to assess its capacity to reproduce the evolution over time of the population distribution. For this, we plot in Figure 2 the visual predictive check (VPC) for the antibody concentration and the ED_{50}^{ν} for all the VoCs. Briefly, the VPC compare the evolution in time of some population percentiles directly estimated from the data with the same quantities reconstructed from the proposed model. The chosen percentiles are the 10th, 50th and 90th ones to assess in a synthetic way if the model is able to reproduce the central population evolution as well as to account for the observed dispersion around it. In our case, the 50th percentile is nearly always well predicted, indicating our model properly describes the median population evolution for Ab and all ED_{50}^{ν}. However, some discrepancies appear between the predicted and empirical 10th and 90th percentiles. Interestingly, these discrepancies always reflect an overestimation of the dispersion around the central trend. Still, this committed error is limited and is less marked for the latest emerging VoCs.

Appendix D: model prediction for different vaccination strategy

To test the external validity of our model, we assess its capacity to make consistent predictions for a cohort presented in [5–7] where, similarly as in our case, antibody concentration are measured in BAU/ml. This cohort originally composed of N=151 participants is divided into two subgroups differing by their age, one of N=89 healthcare workers (HCW)s and a second one of N=62 older peoples (OP)s. Their received up to three doses of mRNA vaccines (either BNT162b2 or mRNA-1273), despite differences in the injection schedules between HCWs and OPs, they both received their second injection later than in our analyzed cohort. Of note, measurements were not available for the same subjects after second and third dose, a summary of measured patient characteristics is given in Table 1.

Antibody concentration are then measured at different timepoints, it is important to note that they are restricted to HCWs prior to the third dose and given for the whole cohort afterward. Regarding predictions, we choose the presented estimated parameter values and we change the injection timing accordingly to this new cohort. Prior to third injection, we took the median injection time among HCWs (i.e. 97 days). For predicting the third dose effect, we took the averaged median injection times for HCWs and OPs weighted by the number of subjects among these subgroups. Measurement and

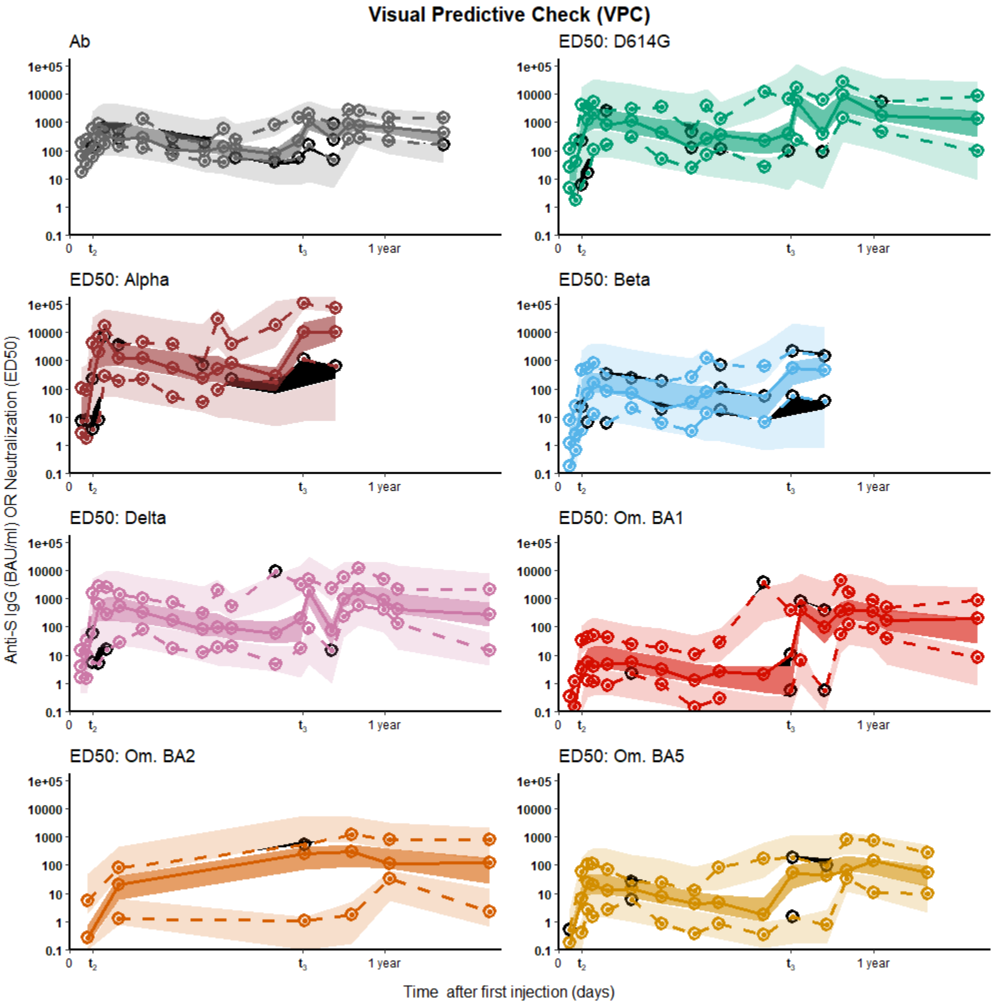

Fig 2. VPC for Ab and ED_{50}^{ν} for the retained model and parametric estimation. The solid lines represent the empirically estimated median and the shaded area the corresponding model predictions when accounting for estimation uncertainty (quantified here by the 90% prediction interval). The dotted lines represent the 10th and 90th percentiles and the faded area the corresponding model predictions. The dots circled in black are the percentiles wrongly predicted by the model. The black area represents the discrepancy between the model prediction and the empirical distributions in case of disagreement.

	Measurements	Model predictions	
		Initial estimation	$f_{\overline{M_3}} := 3.5 f_{\overline{M_3}}$
One month after first dose	1.9	1.8 [1.7; 2.0]	
One month after second dose	2.8	2.7 [2.5; 2.9]	-
Two months after second dose	2.5	2.6 [2.4; 2.7]	-
One month after third dose	3.7	3.3 [3.1; 3.5]	3.7 [3.5; 3.8]
Three months after third dose	3.3	3.0 [2.7; 3.1]	3.3 [3.2; 3.4]
Six months after third dose	2.8	2.4 [2.3; 2.6]	2.9 [2.7; 2.9]

Table 2. Comparison between measurements and model predictions (both given in $\log_{10}(BAU/ml)$. The first column presents the raw measurements. The second column presents the predictions made from the estimations presented in the main article. The third column presents the predictions made with the updated parameter $f_{\overline{M_3}}$.

predictions are presented in Figure 3 and Table 2.

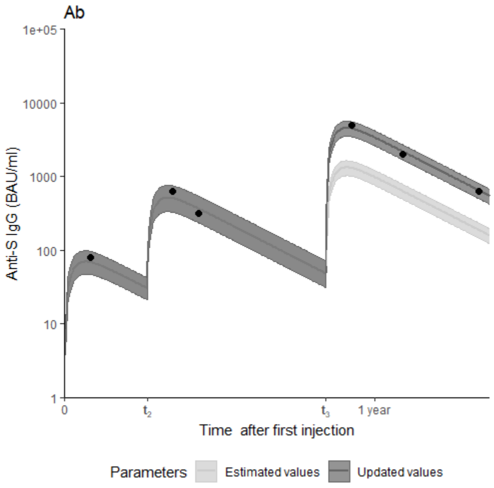

Fig 3. Comparison between measurements and model predictions. The dot are the raw measurements. The light-grey line is the predicted antibody concentration made from the estimations presented in the main article. The dark-grey line is the predicted antibody concentration made with the updated parameter $f_{\overline{M_3}} := 3.5\widehat{f_{\overline{M_3}}}$. Both predictions overlap before the third dose. The shaded area are 95% predictions interval accounting for parameter estimation uncertainty.

While the model correctly predicts the antibody level post first and second dose, it slightly under-estimates antibody level after the third dose. This could be simply due to random fluctuations and measurement error but could also reveal genuine immunological mechanisms that are not accounted for in the model. In particular, nearly half of the subjects in the Canadian cohort received different vaccines, which may induce greater immunogenicity [8]. It is also possible that the timing of administration results in a different maturation kinetics Additionally, the injection timing itself can have an impact on the antibody concentration [9] Still, no matter the underlying cause of the observed differences, updating only one parameter, here $f_{\overline{M_3}}$ ruling the antibody peak after the third dose is enough to re-establish consistency with the last three measurements. This is illustrated in Figure 3 representing the antibody concentration evolution corresponding to the new parameters value $f_{\overline{M_3}} := 3.5\widehat{f_{\overline{M_3}}}$. This illustrates our model capacity to account for other vaccination strategies.

Appendix E: induced neutralization by a fourth dose

Our proposed model can be easily modified to account for the effect of additional doses, in particular a fourth one, and predict the duration of acquired neutralization. This requires to extend our neutralization model now given by:

$$F(\nu, t) = \gamma f_\nu (\mathbb{1}_{t<\mathbf{t_2}} + f_2 \mathbb{1}_{t\in[\mathbf{t_2};\mathbf{t_3}]} + f_3 g_\nu \mathbb{1}_{t\in[\mathbf{t_3};\mathbf{t_4}]} + f_4 g_{4,\nu} \mathbb{1}_{t\geq\mathbf{t_4}})$$

where $\mathbf{t_4}$ is the time of fourth injection, chosen here to be 1 year after $\mathbf{t_3}$. This model definition requires new parameters, f_4 the fold-change for neutralization gain brought by a fourth injection for D614G and $g_{4,\nu}$ the relative VoC-specific gain compared to D614G. In addition to neutralization parameters, we also have to add $f_{\overline{M_4}} = \frac{\overline{M_4}}{\overline{M_1}}$, the fold-change for the memory compartment at $\mathbf{t_4}$ to account for change in Ab dynamics in ODE (3).

Now, different hypotheses on the fourth dose induced humoral response can be translated as hypotheses on $(f_4, g_{4,\nu}, f_{\overline{M_4}})$ values and the proposed model is then used to predict the related neutralization longevity for the different VoCs. In the following we assume that $g_{4,\nu} = g_\nu$, that is, the relative differences in VoC fold-change stays the same from the third to the fourth dose. All tested scenarios then boil down to the choices of $(f_4, f_{\overline{M_4}})$. We investigate four cases which quantify change in humoral response at $\mathbf{t_4}$ comparing to $\mathbf{t_3}$.

Model A - No change : Same affinity and Ab level: $f_4 = f_3$ & $f_{\overline{M_4}} = f_{\overline{M_3}}$.

Model B - Ab change : Same affinity and increased Ab : $f_4 = f_3$ & $f_{\overline{M_4}} = 2.88 f_{\overline{M_3}}$.

Model C - Affinity change : Increased affinity and same Ab : $f_4 = 2f_3$ & $f_{\overline{M_4}} = f_{\overline{M_3}}$.

Model D - Both change : Increased affinity and increased Ab : $f_4 = 2f_3$ & $f_{\overline{M_4}} = 2.88 f_{\overline{M_3}}$.

The retained values quantifying increase have been chosen accordingly to previous estimations such that: $2.88 = \frac{f_{\overline{M_4}}}{f_{\overline{M_3}}} = \frac{f_{\overline{M_3}}}{f_{\overline{M_2}}}$ and $2 = \frac{f_4}{f_3} = \frac{f_3}{f_2}$. The point was to consider increase between the fourth and third injection consistent with the ones inferred the between third and second dose.

Predicted Ab and ED_{50}^{ν} evolution up to 1500 days after $\mathbf{t_1}$ for all considered scenarios are given in Figure 4 . The time required for ED_{50}^{ν} to be undetectable again after $\mathbf{t_4}$ is given in Table 3. We end up with predictions sensitive to assumed parameter values, especially to $f_{\overline{M_4}}$ variation. For example, the time to return to undetectability move from 183 (95% PI ([153; 213]) to 596 (95% PI ([564; 629]) days for BA.5 from the most pessimistic to the more optimistic scenario regarding the triggered humoral response at $\mathbf{t_4}$. Still, we can conclude that if the fourth dose induces an increase for the humoral response similar in proportion to the one observed at $\mathbf{t_3}$, vaccination can induce a neutralization against the dominant VoC detectable almost up to two years.

Table 3. Time to return to undetectability after $\mathbf{t_4}$ (days) with 95% Prediction intervals.

	Relative change w.r.t $\mathbf{t_3}$			
	Model A	Model B	Model C	Model D
	No change	Ab change	Affinity change	Both change
	$f_4 = f_3$		$f_4 = 2f_3$	
	$f_{\overline{M_4}} = f_{\overline{M_3}}$	$f_{\overline{M_4}} = 2.88 f_{\overline{M_3}}$	$f_{\overline{M_4}} = f_{\overline{M_3}}$	$f_{\overline{M_4}} = 2.88 f_{\overline{M_3}}$
D614G	438 [408; 469]	824 [771; 867]	489 [459; 520]	867 [822; 867]
Alpha	587 [538; 634]	867 [867; 867]	638 [589; 685]	867 [867; 867]
Beta	347 [307; 390]	734 [673; 792]	398 [358; 441]	785 [724; 843]
Delta	355 [325; 383]	742 [690; 792]	406 [376; 434]	793 [741; 843]
BA.1	256 [226; 285]	643 [588; 696]	307 [277; 336]	694 [639; 748]
BA.2	243 [207; 274]	629 [569; 688]	294 [258; 325]	680 [620; 739]
BA.5	172 [147; 201]	559 [509; 613]	223 [198; 252]	610 [560; 664]

References

1. Balelli I, Pasin C, Prague M, Crauste F, Thiébaut R. A model for establishment, maintenance and reactivation of the immune response after two-dose vaccination regimens against Ebola virus. Journal of Theoretical Biology. 2020; p. 110254.

2. Castro M, de Boer RJ. Testing structural identifiability by a simple scaling method. bioRxiv. 2020;doi:10.1101/2020.02.04.933630.

3. Muecksch F, Wang Z, Cho A, Gaebler C, Tanfous TB, DaSilva J, et al. Increased potency and breadth of SARS-CoV-2 neutralizing antibodies after a third mRNA vaccine dose. bioRxiv. 2022;.

4. Wang K, Jia Z, Bao L, Wang L, Cao L, Chi H, et al. Memory B cell repertoire from triple vaccinees against diverse SARS-CoV-2 variants. Nature. 2022;603(7903):919–925.

5. Brockman MA, Mwimanzi F, Lapointe HR, Sang Y, Agafitei O, Cheung PK, et al. Reduced magnitude and durability of humoral immune responses to COVID-19 mRNA vaccines among older adults. The Journal of Infectious Diseases. 2022;225(7):1129–1140.

6. Lapointe HR, Mwimanzi F, Cheung PK, Sang Y, Yaseen F, Kalikawe R, et al. Serial infection with SARS-CoV-2 Omicron BA. 1 and BA. 2 following three-dose COVID-19 vaccination. medRxiv. 2022;.

7. Mwimanzi F, Lapointe HR, Cheung PK, Sang Y, Yaseen F, Umviligihozo G, et al. Older adults mount less durable humoral responses to two doses of COVID-19 mRNA vaccine, but strong initial responses to a third dose. medRxiv. 2022;.

8. Naito T, Tsuchida N, Kusunoki S, Kaneko Y, Tobita M, Hori S, et al. Reactogenicity and immunogenicity of BNT162b2 or mRNA-1273 COVID-19 booster vaccinations after two doses of BNT162b2 among healthcare workers in Japan: A prospective observational study. Expert Review of Vaccines. 2022;21(9):1319–1329.

9. Stolfi P, Castiglione F, Mastrostefano E, Di Biase I, Di Biase S, Palmieri G, et al. In-silico evaluation of adenoviral COVID-19 vaccination protocols: Assessment of immunological memory up to 6 months after the third dose. Frontiers in Immunology. 2022;13.

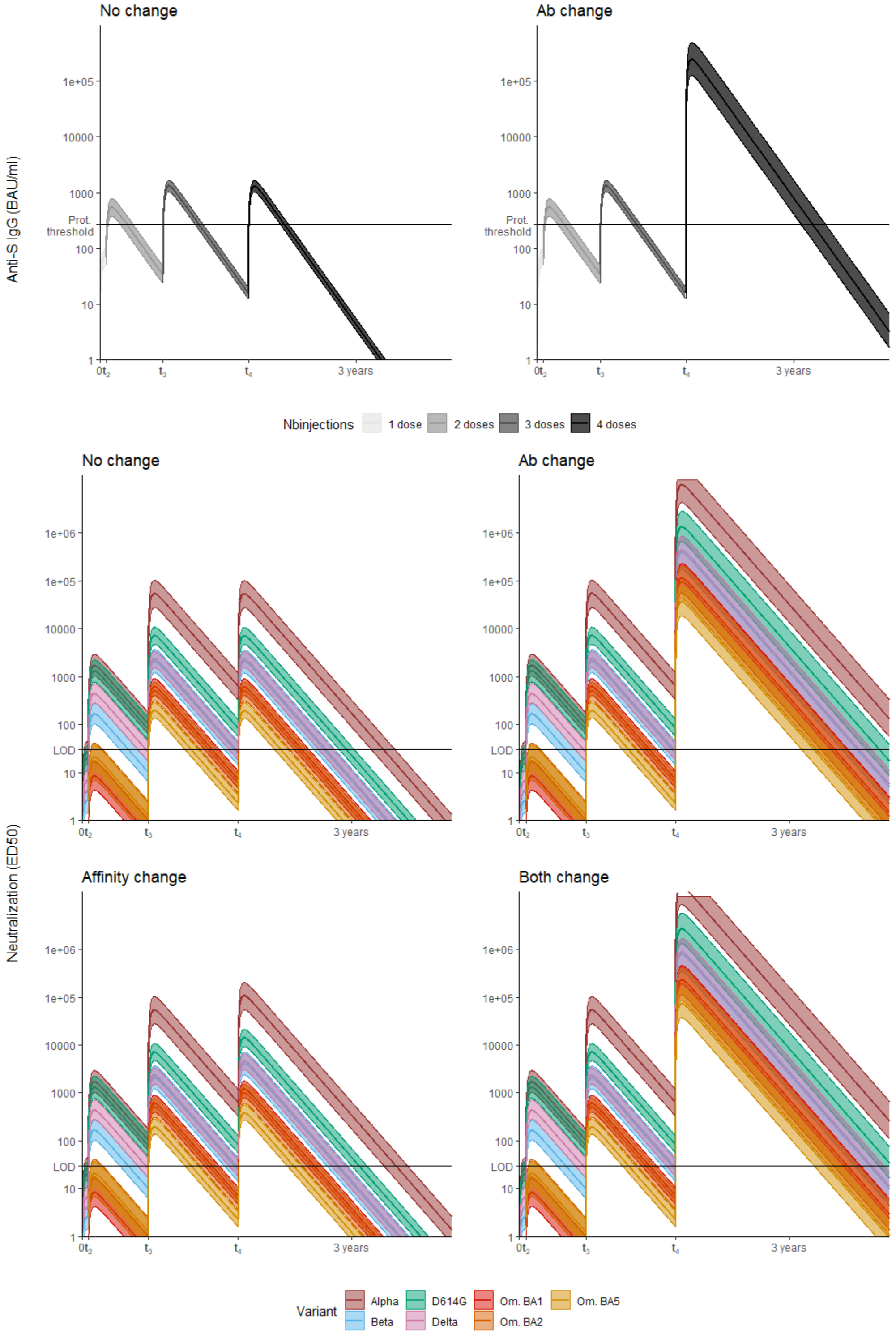

Fig 4. Predicted humoral response for a 4-doses vaccine regimen. Top: Antibody concentration when $f_{\overline{M_4}} = f_{\overline{M_3}}$ (left) and $f_{\overline{M_4}} = 2.88 f_{\overline{M_3}}$ (right). Bottom: Predicted ED_{50}^{ν} when $f_4 = f_3$ and $f_{\overline{M_4}} = f_{\overline{M_3}}$ (top-left); when $f_4 = f_3$ and $f_{\overline{M_4}} = 2.88 f_{\overline{M_3}}$ (top-right); when $f_4 = 2f_3$ and $f_{\overline{M_4}} = f_{\overline{M_3}}$ (bottom-left); when $f_4 = 2f_3$ and $f_{\overline{M_4}} = 2.88 f_{\overline{M_3}}$ (bottom-right).
