# Supplementary material for: Modeling the kinetics of the neutralizing antibody response against SARS-CoV-2 variants after several administrations of Bnt162b2
Source: PLoS Comput Biol. 2023 Aug 7;19(8):e1011282. doi: 10.1371/journal.pcbi.1011282 (PMC10434962; doi:10.1371/journal.pcbi.1011282)
Supplement: S1 File — The dataset used for this analysis is available in the zip file Neutralization_Data_and_code. (ZIP) [file pcbi.1011282.s005.zip › Neutralization_Data_and_code/Readme.docx]

This folder contains three main elements:

1. The formatted database (file “Data_AntibodyConcentration_Neutralization.txt”).
2. The Monolix file and estimation results (file MonolixFile_MemoryModel_NeutralizationModel.mlxtran and folder MonolixFile_MemoryModel_NeutralizationModel).
3. The R file used to reconstruct graphs presented in Section Results (files Construction_Graphs_Ab_ED50_individual_fits.R, Construction_Graphs_ED50_Ab_Tdect and Construction_Graphs_ED50_Ab_4thdose.R).

**I Database architecture**

This database contains different columns defined as such:

1. id: anonymized identifier for each subject (integer ranging from 1 to 26).
2. Time: elapsed time since first injection (in day).
3. SecondInjTime: time of second injection after the first one (in day).
4. ThirdInjTime: time of third injection after the first one (in day).
5. ED50_BAU: log10 transformed measurement of antibody concentration and ED50. The type of measure is given in columns BAU_Variant.
6. BAU_Variant: integer ranging from 1 to 8 specifying the datatype given in the same row for ED50_BAU:
   1. log10(Ab)
   2. log10(ED50_D614G)
   3. log10(ED50_Alpha)
   4. log10(ED50_Beta)
   5. log10(ED50_Delta)
   6. log10(ED50_OmicronBA1)
   7. log10(ED50_OmicronBA2)
   8. log10(ED50_OmicronBA5).
7. Censored: specify if the measurement point was left-censored that is, below the detection level (1 for censored, 0 otherwise). The threshold was set to:
   1. log10(6) for log10(Ab)
   2. log10(7.5) for log10(ED50_D614G), log10(ED50_Alpha), log10(ED50_Beta), log10(ED50_Delta)
   3. log10(30) for log10(ED50_OmicronBA1), log10(ED50_OmicronBA2), log10(ED50_OmicronBA5).
8. Nbinj: precize the dose number received by the subject.

**II Monolix files for parameter estimation**

The Monolix project: MonolixFile_MemoryModel_NeutralizationModel.mlxtran contains everything required to obtain parameter estimation results presented in Table 2. The parametrization used for estimation was slightly different than the one used for results presentation, but link between the two is biunivocal:

1. log_theta_muS_M = ln(\vartheta)
2. log_fc_muS_M2 = ln(\f_M2)
3. log_fc_muS_M3 = ln(\f_M3)
4. log_gamma_D614G = ln(\gamma)
5. log_gamma_nu = ln(f_\nu)
6. log_diff_gamma_D614G_secondinj = ln(f_2)
7. log_diff_gamma_D614G_thirdinj = ln(f_3)
8. log_diff_gamma_nu_thirdinj = ln(g_\nu).

The folder MonolixFile_MemoryModel_NeutralizationModel contains every estimation results, (population parameter, subject specific parameters and Fisher Information Matrix) required to construct graphs presented in Section Results and Appendix C.

**III R scripts for Graphs reconstruction**

The scripts:

1. Construction_Graphs_Ab_ED50_individual_fits.R is used to construct the graphs appearing in Fig 4 and Fig 5. Running time is a matter of hour in a personal computer
2. Construction_Graphs_ED50_Ab_individual_fits.R: is used to construct the individual fits. Running time is a matter of minute in a personal computer.
3. Construction_Graphs_ED50_Ab_4thdose.R: is used to construct the graphs appearing in Appendix C in the counterfactual event where subjects receive an additional fourth dose. Running time is a matter of minute in a personal computer.

All scripts require only to set the folder Plos_Comp_Neutralization_Data_and_code as the working directory to be executed. The script Construction_Graphs_ED50_Ab_individual_fits.R: requires to set the parameter index_id_cur to choose the subject for which we want the individual fits.
