# Supplementary material for: Modeling the kinetics of the neutralizing antibody response against SARS-CoV-2 variants after several administrations of Bnt162b2
Source: PLoS Comput Biol. 2023 Aug 7;19(8):e1011282. doi: 10.1371/journal.pcbi.1011282 (PMC10434962; doi:10.1371/journal.pcbi.1011282)
Supplement: Attachment — Submitted filename: ResponseToReviewers.pdf [file pcbi.1011282.s006.pdf]

1 Responses to reviewers

We thank the reviewers for their insightful comments. Per their comments the following main changes have been made:

1. additional assessment of the model and comparison with other alternative models
2. discussion/comparison with data from the literature, including data obtained with alternative schedules of administrations.

2 Reviewer #1

In this paper, Clairon et al. describe the coupling of a simple, mechanistic model of antibody production from B cells following mRNA vaccination against COVID-19 with a model of neutralization to understand the efficacy of mRNA COVID-19 vaccines against infection with respect to variants of concern. The authors use a simplified expression of their model to derive a piecewise description following multiple doses (primarily 3 doses of the original Wuhan strain vaccine, with some discussion of a 4th dose) of vaccine to understand the durability of neutralization over time. To parameterize their model, they use longitudinal data from a vaccine clinical trial in France and a non-linear mixed effects model (fit in Monolix). Their results indicate that third doses provide the highest neutralization and that Omicron is much less neutralized than other VOCs.

Thank you for the careful reading of the paper. Please find our answers to your comments below.

2.1 Major Comments

1) Understandably, the authors make use of the data that are available to them. In this case, this means a clinical trial where second doses were administered about 30 days after the first. However, certain countries (notably the UK, Canada, etc.) did not administer second doses according to this schedule, thereby affecting antibody titers in their populations over time. It would be interesting to use data from these countries as a validation/discovery substudy. What model parameters are altered in this case? Further, have the authors considered any validation beyond model fitting? This would help to convince on the predictability of the model beyond the individual time courses to which it is fit.

We agree that a discussion of the model predictions with different designs was lacking. To address this question we now compare the model predictions with those obtained in a Canadian cohort where the second dose is administered on average 97 days after the first one [3, 8]. A delay between injection significantly longer than the 27 days observed in the original cohort. These results have been added in Appendix D. We focus our comparison on antibody concentration

evolution since it is the only quantity for which a common unit (BAU/ml) is adopted among the different cohorts.

[Figure 1 about here.]

While the model correctly predicts the antibody level post first and second dose, it slightly under-estimates antibody level after the third dose (see Figure 1 presented in Appendix D). This discrepancy could reveal a limitation of our model, but may also simply be due to limitation in our dataset due to limited population sample, as stated in the section "Discussion": *"Also, despite its simplicity, the estimated mechanistic model for antibody kinetics produces consistent prediction for patient with a different vaccination schedule than the one considered here (see Appendix D in S1 Appendix)".*

A discussion completed in Appendix D by: *"While the model correctly predicts the antibody level post first and second dose, it slightly under-estimates antibody level after the third dose. This could be simply due to random fluctuations and measurement error but could also reveal genuine immunological mechanisms that are not accounted for in the model. In particular, nearly half of the subjects in the Canadian cohort received different vaccines, which may induce greater immunogenicity [10]. It is also possible that the timing of administration results in a different maturation kinetics Additionally, the injection timing itself can have an impact on the antibody concentration [12]. "*

Also, as pointed by the reviewer, validity of the model can also be evaluated by internal evaluation. A standard tool for internal evaluation is the examination of the Visual Predictive Check (VPC), that simultaneously evaluates the structural and the biological components of the model. As can be seeen below, the VPC shows that the model well captures the kinetics observed and its variability across individuals. The VPC has been added in Appendix C and the following sentences have been added in the section "Results": *"To assess the capability of the model to fit our data, we also examined the visual predictive check (see Appendix C), which showed that the model well captures the kinetics observed and its variability across individuals."*

[Figure 2 about here.]

2) I have concerns about the model simplification used to address the model identifiability (and importantly, I believe to facilitate parameter estimation). As pointed out in the SI, the value $\bar{M}_k$ is the achieved equilibrium value. However, particularly for a low number of doses (i.e., 1-3), the behaviour of the model is still transient and thus how relevant or valid is it to use a steady-state-state assumption? Monolix allows for multiple doses so I'm not sure why this approach was used vs multiple doses in the dataset (one can see that the piecewise function was used for dosing in the attached code).

The reviewer is correct that approximating M by its steady-state value $\overline{M_k}$ was made to ensure model identifiability. Per the reviewer comment we now discuss the statistical and mathematical validity of this approximation in section "Mechanistic model for antibody kinetics": *"Of note, we also tested the*

full model which does not assume a steady state value for M. This leads to identifiability issues mainly due to μ_S estimation for which only a lower bound ($\mu_S > 20$) can be found. For such values for μ_S, the compartment M nearly instantaneously reaches its steady-state. Accordingly, both full and simplified models provide virtually similar predictions for Ab (see Appendix A.2 in S1 Appendix)." The technical details supporting this discussion are given in Appendix A.2. In particular, we plot in figure (3)-right the predicted Ab trajectories for μ_S ranging from 20 to 200 and the only corresponding prediction from the simplified ODE.

[Figure 3 about here.]

3) The mechanistic model of antibody production is quite simplified and relies on ODEs. However, it is known that there are delays in the processes of both generating antibody-producing B cells (here collapsed into one population M) and antibody from these cells. Since this model does not seem to take this into account, what are the implications of this modelling choice? It seems intuitively that they could be significant. This is somewhat discussed on page 5 line 95 but predictions are not shown and the discussion there does not relate specifically to this issue.

The reviewer is correct that our model may over-predict antibody growth right after each injection. To quantify this phenomenon, we now also test a model with a compartment between M and S to account for latency between generation and differentiation. As can be seen Appendix A.2, this model leads to a deterioration of the AIC, due to overparameterization. Indeed, when restricted to antibody concentration data, parameter estimation with this new model leads to an AIC of 17.6, higher than AIC=16.5 obtained for the retained ODE. However, it is possible that such model could be relevant if more data were available early after dose injection. This is now discussed in section "Mechanistic model for antibody kinetics": *"Finally, we also tested a more complex model accounting for a delay between vaccine injection and antibody production (see Appendix A.2). However the model did not improve data description, which was probably due to the limited amount of information available on antibody kinetics in the couple of days following vaccine injection."* As for the previous point, the technical details supporting this discussion are left in Appendix A.2.

4) Further, the neutralization function used is linear instead of sigmoidal. In the SI, the authors discuss the AICs of each of their neutralization function hypotheses. Perhaps I've misunderstood their analysis, but why is the lowest AIC (H2) not chosen? Nonetheless, experimental data shows that patient-derived antibodies and monoclonal antibodies neutralize SARS-CoV-2 with a sigmoidal relationship and therefore the choice of neutralization function should be dictated by them and not a statistical argument, in my view.

First of all, we would like to thank you for noticing a typo in the submitted manuscript, where the correct value for AIC was 1779 (and not 179). Please note that in the current manucript, the AIC value has changed to 1747 due to the fact that an additional parameter is now estimated (see comment 5). Although a sigmoid model was not supported by the data available in this study,

we agree that such model is likely more physiological, and we now discuss this point as follows in section "Functional model for neutralizing activity": *Our model assumes a simple linear relationship between antibody concentration and neutralization, with no saturation effect. The fact that a more physiological model assuming a nonlinear relationship did not improve data description (see Appendix B) suggests that the level of Antibody observed in this study remains within the linear range of neutralization.*

5) The clearance rate of antibodies was fixed–couldn't this have a significant impact on the antibody kinetics being predicted?

Per your comment, we have now allowed the parameter δ_{Ab} to be estimated. This indeed slightly improved data description, and led to an estimated value for δ_{Ab} corresponds approximately to an half life of 9 days, which is close to the typical range of 10-21 days [2]. This is discussed as follows in section "Results": *Of note, the estimated value for δ_{Ab} approximately corresponds to an half-life of 9 days, which is close to the typical range of 10 and 21 days [2].* All models have been updated accordingly to ensure fair comparison between the models, and all results have been updated in the revised manuscript.

2.2 Minor Comments

1) As the authors note in the discussion, effective neutralization and detectable neutralization (i.e., the difference between assay-based measurements of neutralization and real-world protection from infection) are different. Perhaps some care should be taken in the abstract with respect to the description of the findings.

The abstract has been modified accordingly. We replaced "protection" by "neutralization". We also carefully proofread the article to avoid this confusion elsewhere.

2) There are a few typos throughout. Notably COVID-19 (all uppercase) and Covid-19 are both used and SARS-CoV-2 virus does not need the "virus" after the acronym. Adjustements should be Adjustments, for example.

Corrected.

3 Reviewer #2

This is a very nice paper on modeling the evolution of neutralizing antibody responses to SARS-CoV-2 variants following the administration of multiple doses of the Bnt162b2 vaccine. The paper simultaneously considers data of antibody concentrations (IgG levels) and their neutralizing ability (ED50) against all the major variants of concern following the administration of a single, two, and three doses of the vaccine. This is thus a rich and timely dataset, given the continued emergence of new variants. The authors develop a modeling framework that considers the dynamics of memory B cell formation and antibody production following vaccination. The data suggest that the IgG levels and ED50 levels are linked in non-trivial ways with the relationship between the two being different

after each dose and with each variant. To recapitulate this observation, the authors use phenomenological constructs that link the IgG and ED50 levels after each dose and with each variants. They estimate these linking parameters using a mixed-effects modeling approach. The model seems to capture the antibody dynamics in individuals well. They then apply the model to predict the durability of the protective antibody response following one, two and three doses, for each of the variants. The predictions reinforce the prevalent notion that with some of the omicron variants the protection is not only limited but is also likely to wane soon after vaccination. The framework developed is useful and is likely to help analyze new data that may arise following additional vaccine doses or the emergence of new variants. The analysis is rigorous and the paper is well written. I have a few minor comments for the authors to consider.

Thank you for your very positive comments and the important points raised in your review, they are adress below.

3.1 Major Comments

1) An important result from the study is that following the second or third dose, the neutralization capacity of antibodies 'per capita' increases compared to the previous dose (first or second) in most cases. This gives hope that with each subsequent dose, the protection will not only improve in quantity but also in quality, and thus become increasingly durable. While the model does well to recapitulate this observation using phenomenological constructs, the authors do not discuss possible mechanistic origins of this observation. A plausible explanation lies in the ongoing affinity maturation in germinal centres or the seeding of germinal centres with memory cells from previous doses. A simulation study explored these possibilities to explain the improvement in protection following delayed boosting [4]. The authors may wish to discuss this and any other hypotheses based on their data that they feel may offer some insights into the observation. Would, for instance, comparing the improvement in per capita neutralization ability following the third versus second dose be consistent with greater affinity maturation given the longer interval between the second and third doses compared to the first and second? This may also help understand why the function F linking ED50 and IgG levels must depend explicitly on time.

Thank you for mentioning the affinity maturation process in germinal center as a plausible origin of the neutralization gain. Regarding the higher neutralization gain observed after the third injection compared with the second one, Garg et al. [4] indeed provides a consistent explanation, with a longer delay between injection increasing the selection stringency in germinal centers. Although such mechanisms could not be evaluated due to data limitation, their effects are consistent with measurements made on other cohorts with alternative vaccination schedule. For example, this is the case for a Canadian cohort described in [3, 8] with a significantly longer delay between injections comparing to ours. For this cohort, the measured antibody concentration after third injection was significantly higher than our prediction (see Appendix D) . We now discuss this in section "Discussion" as follows: *"Our model does not inte-*

grate the possible mechanisms causing the gain in neutralization observed over dose injections, and how this may modulated by the time between injections. For instance, it has been suggested that longer delay between injections could increase the Memory B-cells selection stringency in germinal centers [4]. This could in turn lead to strategies to maximize antibody concentration, as suggested by theoretical models [12]. This is in line with measurements made on another cohorts, for example the one described in [3, 8] with a significantly longer delay between injections comparing to ours. In this case, measured antibody concentration after third injection was significantly higher than our prediction. Still, due to our data limitation (few subjects with similar vaccination schedule), we cannot isolate and thus estimate the effect of injection schedule on neutralizing activity."

Still, on a purely formal point of view, the effect of injection schedule can be integrated in the parameters or model definition. So, if more informative datasets become available we can hope to estimate such schedule effect. That is why we continue the discussion as follows: *"In the future, application of such approaches to larger populations of individuals, with a wider range of tested vaccination schedules, may allow some of these hypotheses to be relaxed and injection timing to be integrated into the model specification without compromising the identifiability of the parameters."*

2) The authors cite the paper by [7], but do not discuss the similarities or differences between their findings and those of the present study. I feel that this would be a useful discussion for the readers.

Korosec et al. [7] is indeed relevant as another modeling exercise of immune response focusing on the interplay between T-cells and B-cells response. Comparisons between the two models are difficult, as they did not integrate neutralization, which is the main focus of our work. However we now mention this reference in section "Discussion" within a broader discussion about the model extensions which now reads: *"Due to the available data and constructed model, we restrict our analysis to the humoral part of the immune response triggered by vaccination only. Still, vaccination also induces a cellular immune response which may contribute to the clinical protection especially against the VoCs [9] (for a mechanistic model accounting for T-cell response, see Korosec et al. [7])."*

Still, on the restricted problem of Ab evolution after second injection, both our work and Korosec et al. [7] can make predictions, but they are significantly different. From their estimated mechanistic model, they estimate t_α, the elapsed time after second injection at which the remaining antibody only represents the fraction $\alpha Ab(t_m)$ of peak value $Ab(t_m)$. In Korosec et al. [7], the t_α' are estimated for values $\alpha = \{0.75, 0.50, 0.25, 0.01\}$ to quantify the speed of waning immunity; the smaller the t_α's are, the quicker the antibody concentration collapses. Estimation from our model and Korosec et al. [7] are given in Table 1, we clearly predict a more sustained humoral response in time. But because of their under-prediction of long term antibody evolution (Figure 2.a in [7]) and in the absence of prediction interval quantifying uncertainty in Korosec et al. [7], it is questionable to draw conclusions from this comparison. It is thus omitted

in the manuscript.

[Table 1 about here.]

3) The authors focus on antibody responses and their neutralization capacity but may wish to comment on other arms of the immune response, particularly CD8 T cells, which may be triggered in response to vaccination. Although these may not have been significant against the ancestral strain, as the antibody efficacies drop against the variants, it could be that the relative roles of the cellular arms become significant. The authors may wish to comment on this possibility, especially since cellular responses as well as innate immune responses have been seen to Bnt162b2 vaccination [9].

That is true that this work omits the evolution of cells and innate immune response. This in accordance with our available data restricted to the measurement of circulating antibodies and their neutralizing activity. We cited the suggested reference and elaborated on this topic in the discussion mentionned in the previous point.

4) The authors construct a phenomenological relationship between antibody levels and ED50. In an interesting modeling study, a mechanistic description of this relationship for the ancestral strain has been developed [11]. The latter study also offers a potential interpretation of inter-patient variability. The authors may wish to mention this study, although its relevance to variants remains to be tested.

This is indeed a very interesting work proposing a consistent and alternative way to link ED50 and antibodies. Still, the definition of their mechanistic relationship involves parameters such as infection events and neutralizing antibody measurements. In our analysis, all of these quantities are VoCs specific and unavailable. This makes their model intractable for our prediction purpose. We now mention this study in the section "Functional model for neutralizing activity": *"Finally, we acknowledge the existence of other ways than our descriptive approach to link Ab and* ED_{50}^{ν}*, as in Padmanabhan et al. [11] in which a mechanistic relationship between these quantities is constructed. Still, their model definition involves measurements, such as infection events or neutralizing antibodies, which are not at our disposal, especially for emerging VoCs, making their model intractable for our prediction purpose."*

3.2 Minor Comments

1) The title indicates modeling the 'evolution' of antibody responses. The study, however, does not really model this evolution explicitly and so appears somewhat misleading.

We replace the term "evolution" by "kinetic".

2) The abstract mentions 'optimal timing' of vaccination. Given the phenomenological model for linking IgG titres with ED50, I am not certain how optimal timings would be arrived at. If this could be done, it would be worth including in the results.

We remove the last sentence in the abstract mentioning the optimal timing.

3) The results of the fourth dose are in the supplementary and are discussed only at the very end of the discussion section. I wonder if these should be mentioned in the results section, especially since the fourth dose may be more widely administered in some countries like Israel than France.

We rather let this analysis in discussion. It is more a proof of concept about our model ability to explore the consequences of different scenarios regarding the efficiency of a fourth dose on neutralization evolution than an actual prediction of the fourth dose effect. Putting this analysis in the results section may be misleading and let the impression we stand behind these numerical results as actual prediction about what will happen for patients who received four doses.

4 Reviewer #3

In this manuscript, Clairon et al. use a mathematical model to understand the temporal dynamics of antibody titres (Ab) and neutralisation capacity against various SARS-CoV-2 variants of concerns (VOCs) following multiple doses of the BNT162b2 vaccine. They employ a model that was previously developed to examine vaccine-induced humoral responses against Ebola infection (PMID: 32205143). The central claim of this study is that the neutralisation capacity for a given antibody concentration increases after successive doses of vaccination. The authors then make predictions of long-term vaccine-induced antibody responses against SARS-CoV-2 VOCs. This study is interesting, timely, and well-conducted. However, there are some minor issues (see below) that would need to be addressed before acceptance.

Thank you for your time and consideration. We appreciate you enthusiastic feedback and addressed the points you have raised.

4.1 Major Comments

1) It would be helpful if the authors could provide more information on the statistical analysis in Figure 2:

Thank you for the improvements you have suggested. Before answering them point by point, we present the modified Figure 2 (here Figure 4) and subsequent discussion in section "Results": *"This is supported by the linear regressions of ED^{ν}_{50} on Ab after each injection presented in Table 2 (made with the R package Censreg [6] to account for censored data). These regressions indicate an increased correlation between ED^{ν}_{50} and Ab with respect to the injection numbers for most of VoCs (already pointed out by Goel et al. [5] for D614G and Beta)."*

[Figure 4 about here.]

[Table 2 about here.]

1a) It needs to be clarified whether they have performed multiple comparison corrections (e.g., Bonferroni correction) and whether they report absolute or corrected p-values.

We now perform Holm correction (uniformly more powerful than Bonferroni correction [1]) to account for multiple comparison and we report the corrected p-values.

1b) It would help if the authors could mention the p-values rather than using asterisks.

Done.

1c) Also, the variance seems to be different across groups, and it is unclear if the data in some groups are normally distributed. It is therefore unclear whether the Student's t-test is appropriate.

We now use Wilcoxon test to bypass the implicit normality hypothesis.

1d) I wonder if the authors could do a linear regression using the expression ED50 = gamma*Ab, estimate gamma at different doses, and show that gamma changes with doses. This might complement the parameter estimation using mixed-effect modelling performed in Figure 3.

We now use the Censreg package [6] to perform linear regressions with censored data, results are presented in Table 2.

2) The authors could consider discussing their work in the context of existing COVID-19 vaccine modelling and experimental studies:

2a) A recent modelling study examined a plausible mechanistic link between antibody levels and ED50 for the original strain, and variations in ED50 amongst vaccinated individuals [11].

This has been pointed out by another reviewer. Still, using for prediction purpose the mechanistic relationship between neutralizing antibody concentration (Nab) and ED50 derived in Padmanabhan et al. [11] requires information (for examples, Nab and infection event measurements) which are not at our disposal in our analysis for VoCs. We now mention this study in the section "Functional model for neutralizing activity": *"Finally, we acknowledge the existence of other ways than our descriptive approach to link Ab and* ED_{50}^{ν}*, as in Padmanabhan et al. [11] in which a mechanistic relationship between these quantities is constructed. Still, their model definition involves measurements, such as infection events or neutralizing antibodies, which are not at our disposal, especially for emerging VoCs, making their model intractable for our prediction purpose."*

2b) Simulation studies have explored how different vaccination protocols affect the quality and quantity of antibody responses following multiple vaccination doses [4, 12].

The reference to Garg et al. [4] had been pointed our by another reviewer. Indeed, they propose a consistent explanation regarding the higher neutralization gain observed after the third injection comparing to the second one. A higher delay between injection can increase the selection stringency in germinal centers and let only Memory B-cells with high affinity survive at the time of next injection. Still due to our data limitation (subjects with similar injection schedule), we cannot isolate and estimate the intrinsic effect of injection delay on neutralization gain. It is also true that the work of Stolfi et al. [12] is in accordance with the antibody concentration level we estimate after third injection despite focusing on Adenovirus-based vaccine. We add a discussion on

the subject in section "Discussion": *"Our model does not integrate the possible mechanisms causing the gain in neutralization observed over dose injections, and how this may modulated by the time between injections. For instance, it has been suggested that longer delay between injections could increase the Memory B-cells selection stringency in germinal centers [4]. This could in turn lead to strategies to maximize antibody concentration, as suggested by theoretical models [12]. This is in line with measurements made on another cohorts, for example the one described in [3, 8] with a significantly longer delay between injections comparing to ours. In this case, measured antibody concentration after third injection was significantly higher than our prediction. Still, due to our data limitation (few subjects with similar vaccination schedule), we cannot isolate and thus estimate the effect of injection schedule on neutralizing activity."*

2c) Goel et al investigated the relationship between antibody titre and FRNT50 (ED50 equivalent) for D614G and B.1.351 before and after booster. They found that the correlation between antibody titre and FRNT50 became stronger after booster [5], suggesting an improvement in the quality of antibody response following booster dose.

We add a mention to this work in section "Results" which shares our conclusion regarding the effect of additional injections on antibody neutralization gain, especially for VoCs: *"These regressions indicate an increased correlation between ED_{50}^{ν} and Ab with respect to the injection numbers for most of VoCs (already pointed out by Goel et al. [5] for D614G and Beta)."*

4.2 Minor Issues

1) Figure 1 (bottom, left panel): It's a bit hard to read the values. I wonder if providing a zoomed-in version as an inset would be helpful.

A zoomed version is provided as a supplementary figure. Still, there is not that much additional informations. This figures shows the neutralizing activity after one dose which is very low for all VoCs. At the exception of D614G, the median values are always under the threshold of detection.

2) Figure 1 (bottom, middle panel): The kinetics of ED50 does seem heterogeneous with time, as mentioned by the authors. Could this effect be simply due to data grouping?

That is a possibility, but other data grouping rules lead to the same observed trend.

3) Ethics statement is a bit confusing because my understanding is that no new data is generated in this study, and all the human subject data is already published elsewhere (Refs. 20 and 21). Is this correct?

That is correct, in this section we just recall the agreement for the cohort constitution and experimental measurements.

4) Typo on line 96; 'no' instead of 'not'?

Corrected.

References

[1] Mikel Aickin and Helen Gensler. Adjusting for multiple testing when reporting research results: the bonferroni vs holm methods. *American journal of public health*, 86(5):726–728, 1996.

[2] Brian J Booth, Boopathy Ramakrishnan, Kristin Narayan, Andrew M Wollacott, Gregory J Babcock, Zachary Shriver, and Karthik Viswanathan. Extending human igg half-life using structure-guided design. In *MAbs*, volume 10, pages 1098–1110. Taylor & Francis, 2018.

[3] Mark A Brockman, Francis Mwimanzi, Hope R Lapointe, Yurou Sang, Olga Agafitei, Peter K Cheung, Siobhan Ennis, Kurtis Ng, Simran Basra, Li Yi Lim, et al. Reduced magnitude and durability of humoral immune responses to covid-19 mrna vaccines among older adults. *The Journal of Infectious Diseases*, 225(7):1129–1140, 2022.

[4] Amar K Garg, Soumya Mittal, Pranesh Padmanabhan, Rajat Desikan, and Narendra M Dixit. Increased b cell selection stringency in germinal centers can explain improved covid-19 vaccine efficacies with low dose prime or delayed boost. *Frontiers in immunology*, page 5064, 2021.

[5] Rishi R Goel, Sokratis A Apostolidis, Mark M Painter, Divij Mathew, Ajinkya Pattekar, Oliva Kuthuru, Sigrid Gouma, Philip Hicks, Wenzhao Meng, Aaron M Rosenfeld, et al. Distinct antibody and memory b cell responses in sars-cov-2 naïve and recovered individuals after mrna vaccination. *Science immunology*, 6(58):eabi6950, 2021.

[6] Arne Henningsen. Estimating censored regression models in r using the censreg package. *R package vignettes*, 5:12, 2010.

[7] Chapin S Korosec, Suzan Farhang-Sardroodi, David W Dick, Sameneh Gholami, Mohammad Sajjad Ghaemi, Iain R Moyles, Morgan Craig, Hsu Kiang Ooi, and Jane M Heffernan. Long-term durability of immune responses to the bnt162b2 and mrna-1273 vaccines based on dosage, age and sex. *Scientific Reports*, 12(1):21232, 2022.

[8] Hope R Lapointe, Francis Mwimanzi, Peter K Cheung, Yurou Sang, Fatima Yaseen, Rebecca Kalikawe, Sneha Datwani, Rachel Waterworth, Gisele Umviligihozo, Siobhan Ennis, et al. Serial infection with sars-cov-2 omicron ba. 1 and ba. 2 following three-dose covid-19 vaccination. *medRxiv*, 2022.

[9] Chunfeng Li, Audrey Lee, Lilit Grigoryan, Prabhu S Arunachalam, Madeleine KD Scott, Meera Trisal, Florian Wimmers, Mrinmoy Sanyal, Payton A Weidenbacher, Yupeng Feng, et al. Mechanisms of innate and adaptive immunity to the pfizer-biontech bnt162b2 vaccine. *Nature Immunology*, 23(4):543–555, 2022.

[10] Toshio Naito, Nao Tsuchida, Susumu Kusunoki, Yoshihiro Kaneko, Morikuni Tobita, Satoshi Hori, and Suminobu Ito. Reactogenicity and immunogenicity of bnt162b2 or mrna-1273 covid-19 booster vaccinations after two doses of bnt162b2 among healthcare workers in japan: A prospective observational study. *Expert Review of Vaccines*, 21(9):1319–1329, 2022.

[11] Pranesh Padmanabhan, Rajat Desikan, and Narendra M Dixit. Modeling how antibody responses may determine the efficacy of covid-19 vaccines. *Nature Computational Science*, 2(2):123–131, 2022.

[12] Paola Stolfi, Filippo Castiglione, Enrico Mastrostefano, Immacolata Di Biase, Sebastiano Di Biase, Gianna Palmieri, and Antonella Prisco. In-silico evaluation of adenoviral covid-19 vaccination protocols: Assessment of immunological memory up to 6 months after the third dose. *Frontiers in Immunology*, 13, 2022.

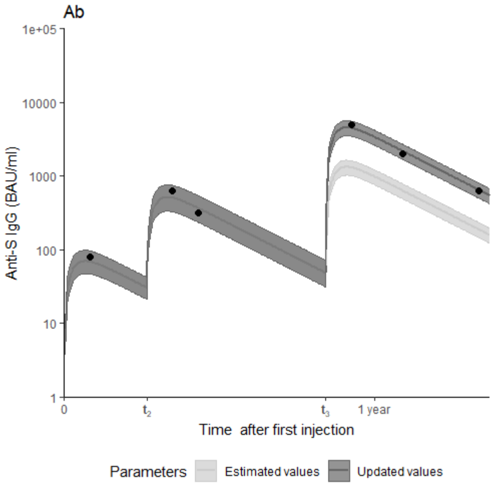

Figure 1: Comparison between measurements and model predictions. The dot are the raw measurements. The light-grey line is the predicted antibody concentration made from the estimations presented in the main article. The dark-grey line is the predicted antibody concentration made with the updated parameter $f_{\overline{M_3}} := 3.5\widehat{f_{\overline{M_3}}}$. Both predictions overlap before the third dose. The shaded area are 95% predictions interval acounting for parameter estimation uncertainty

.

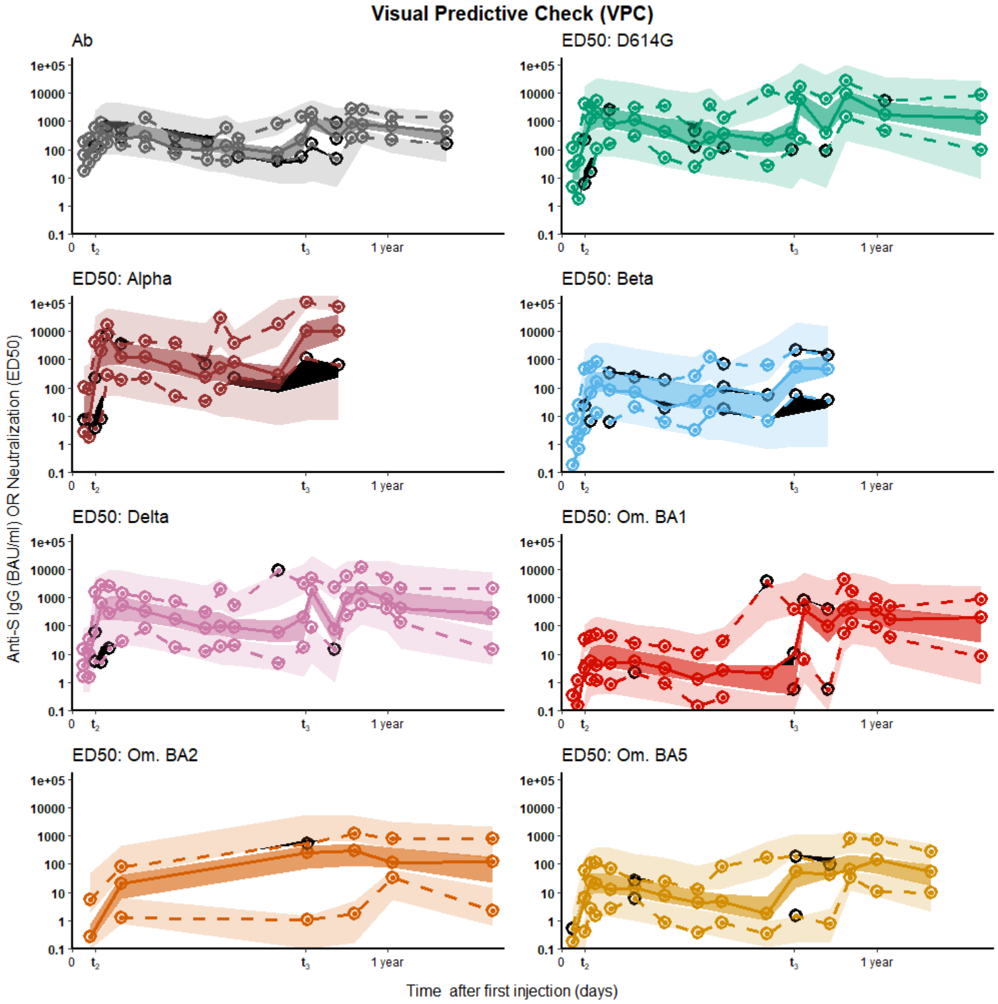

Figure 2: VPC for *Ab* and ED_{50}^{ν} for the retained model and parametric estimation. The solid lines represent the empirically estimated median and the shaded area the corresponding model predictions when accounting for estimation uncertainty (quantified here by the 90% prediction interval). The dotted lines represent the 10th and 90th percentiles and the faded area the corresponding model predictions. The dots circled in black are the percentiles wrongly predicted by the model. The black area represents the discrepancy between the model prediction and the empirical distributions in case of disagreement.

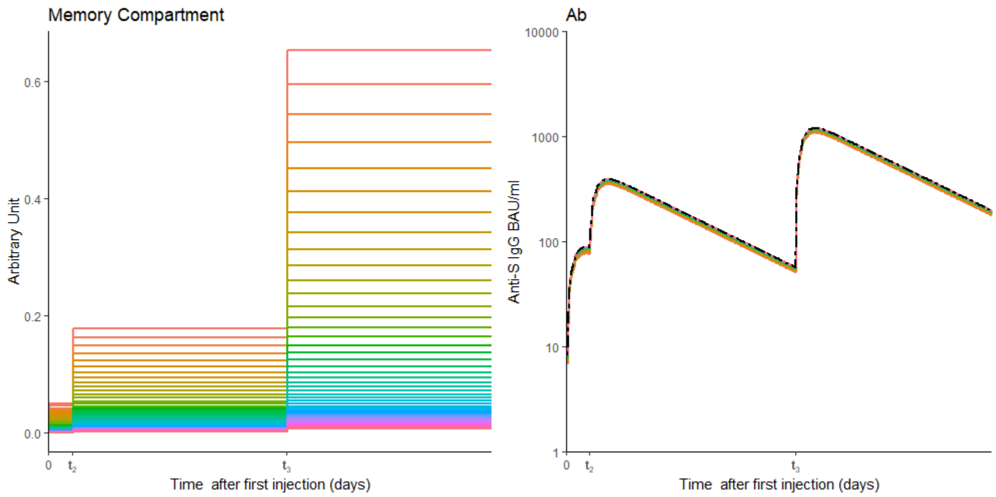

Figure 3: Reconstituted trajectories with ODE accounting for M transient phase for μ_S value ranging from 20 to 200. Left: Memory Compartment M. Right: Antibody concentration, the dashed black line represent the estimated prediction from simplified ODE.

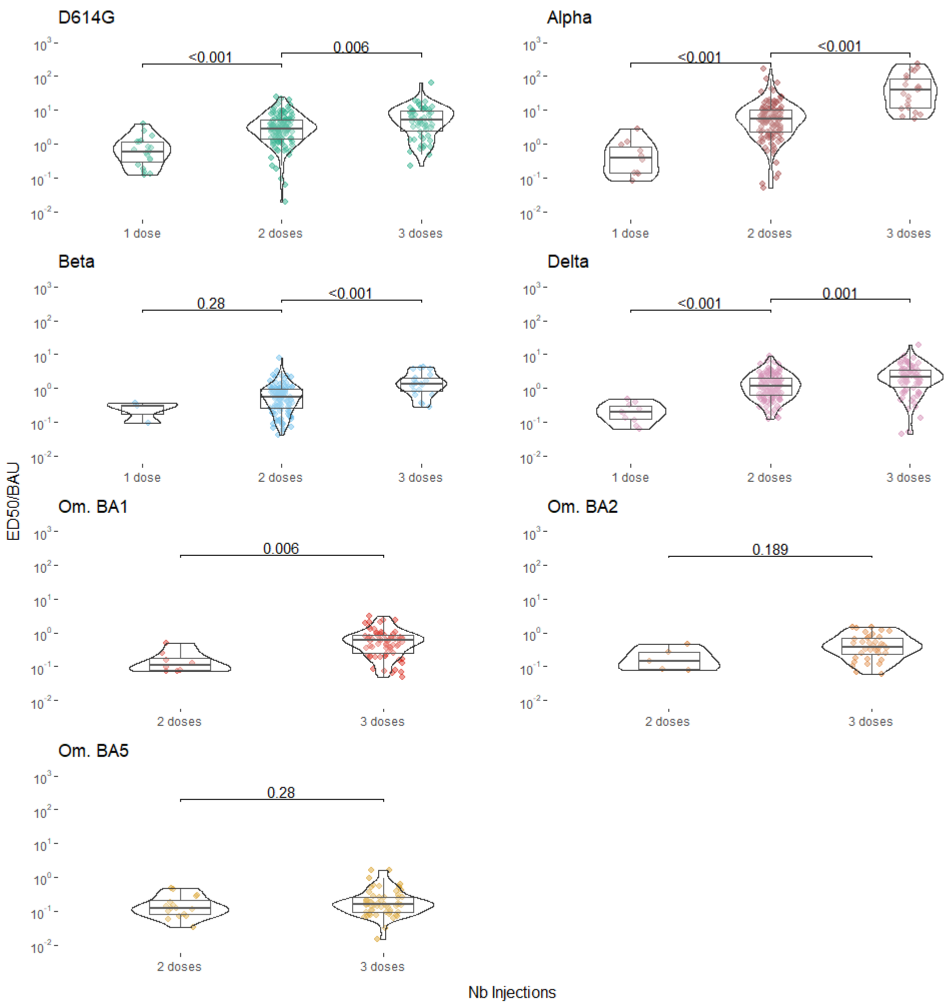

Figure 4: Evolution of the predicted ratio ED_{50}^{ν}/BAU for each VoC after successive vaccine doses. Each circle represents a ratio ED_{50}^{ν}/BAU computed when both measurements for ED_{50}^{ν} and BAU where available for a given patient at a given observation time. Most of patients contribute several times due to the repeated measurements made over time after each dose. All predictions below the limit of detection for ED_{50}^{ν} were removed to avoid overoptimistic ED_{50}^{ν}/BAU ratio when replacing ED_{50}^{ν} values by detection threshold. This explains why very few values are available for Beta and Delta and none for Omicron strains for one dose case. Comparison between vaccine dose were done using Wilcoxon test with Holm correction, p-values are given above the brackets.

Table 1: Waning immunity speed estimation

	Estimation	
α	Korosec et al. [7]	Our article
0.75	72	78 [68; 91]
0.50	88	109 [98; 124]
0.25	110	160 [149; 176]
0.01	190	397 [386; 413]

Table 2: Linear regression results with censored data

	β estimation in model $ED_{50}^{\nu} = \alpha + \beta Ab$ on interval:		
	$[\mathbf{t}_1; \mathbf{t}_2[$	$[\mathbf{t}_2; \mathbf{t}_3[$	$[\mathbf{t}_3; +\infty[$
D614G	0.90 [0.01; 1.73]	2.42 [1.48; 3.47]	15.31 [8.93; 21.68]
Alpha	0.50 [-0.26; 1.24]	4.39 [2.02; 6.76]	38.65 [3.24; 74.06]
Beta	0.10 [-0.15; 0.30]	0.30 [0.15; 0.45]	1.83 [0.93; 2.70]
Delta	2.05 [-10.5; 12.30]	1.58 [1.11; 2.02]	5.86 [4.16; 7.55]
BA.1	-	0.11 [0.01; 0.22]	1.08 [0.74; 1.41]
BA.2	-	0.16 [0.02; 0.30]	0.21 [0.08; 0.36]
BA.5	-	0.15 [0.07; 0.23]	0.51 [0.31; 0.71]
