# Supplementary material for: Modeling the kinetics of the neutralizing antibody response against SARS-CoV-2 variants after several administrations of Bnt162b2
Source: PLoS Comput Biol. 2023 Aug 7;19(8):e1011282. doi: 10.1371/journal.pcbi.1011282 (PMC10434962; doi:10.1371/journal.pcbi.1011282)
Supplement: Attachment — Submitted filename: ResponseToReviewers.pdf [file pcbi.1011282.s007.pdf]

1 Responses to reviewers

We thank the reviewers for their insightful comments. We have added an analysis regarding the linear relationship between binding antibody concentration and neutralizing activity.

2 Reviewer #1

2.1 Major Comments

1) I thank the authors for their revisions. Many of my previous questions were addressed but I still have an issue with the antibody neutralization function. The added statement that "Antibody observed in this study remains within the linear range of neutralization" can be tested through comparison with available antibody neutralization data. Why not test this explicitly through validation to external data?

The analysis of the relationship between binding antibody concentration (given in BAU/ml) and neutralizing activity was indeed restricted to our analyzed cohort. One crucial limitation for external comparisons is the lack of a standardized experimental setting.

With the data available in our cohort, we have fitted each Voc neutralising capacity on antibody concentration, they are presented here in Figure 1. They indicate that linear relationship captures well different sources of ED_{50}^{ν} variation and thus constitutes a relevant and simple model. In terms of modeling, the linear model is then a valuable alternative to more complex ones such as the sigmoid function when this latter does not improve data description as it had been already pointed out and detailed in Appendix B. In the study presented in Lingas et al. [1] using the same protocol than in ours, (and involving some of the authors and this work) a linear relationship was also found to provide a good fit to data in the context of antibody response after infection.

This linear relationship has also been found in other studies based on different experimental protocols regarding antibody concentration/neutralization activity quantification. For instance, in Manenti et al. [2] the linear regression of neutralization against the Wuhan strain on antibody concentration leads to an adjusted R squared of $R^2 > 0.83$ in a cohort of n=57 subjects. This applies as well to the work of Tran et al. [3] in which this linear relationship is also found between antibody concentration and neutralizing activity against VoCs (Omicron inlcuded) in a cohort of more than a thousand of subjects.

In order to add more details in the manuscript on this important note, we add the following passage line 302: *The choice of linear relationship between neutralizing activity and binding antibodies obviously omits features acting on neutralization which could have been incorporated in a more complete model, closer to biological mechanisms. Still, Figure 1 shows that this choice is in adequation with general trend in our data and testing more complex relationships, such as a sigmoid model, did not lead to statistical improvement (see appendix*

B). Of note, this linear relationship also provided a good fit to the data on external cohorts [1, 3, 2].

[Figure 1 about here.]

2.2 Minor Comments

1) Revised title: kinetic should be kinetics

Corrected.

2) "Comparison between vaccine doses were" should be "Comparison between vaccine doses was".

Corrected.

References

[1] Guillaume Lingas, Delphine Planas, Hélène Péré, Darragh Duffy, Isabelle Staropoli, Françoise Porrot, Florence Guivel-Benhassine, Nicolas Chapuis, Camille Gobeaux, David Veyer, et al. Modelling the association between neutralizing antibody levels and sars-cov-2 viral dynamics: implications to define correlates of protection against infection. *medRxiv*, pages 2023–03, 2023.

[2] Alessandro Manenti, Elena Gianchecchi, Francesca Dapporto, Margherita Leonardi, Paolo Cantaloni, Filippo Fattorini, Pietro Piu, Valentina Bollati, Ugo Pastorino, Giovanni Apolone, et al. Evaluation and correlation between sars-cov-2 neutralizing and binding antibodies in convalescent and vaccinated subjects. *Journal of Immunological Methods*, 500:113197, 2022.

[3] Trung The Tran, Eline Benno Vaage, Adi Mehta, Adity Chopra, Lisa Tietze, Anette Kolderup, Aina Anthi, Marton König, Gro Nygaard, Andreas Lind, et al. Titers of antibodies against ancestral sars-cov-2 correlate with levels of neutralizing antibodies to multiple variants. *npj Vaccines*, 7(1):174, 2022.

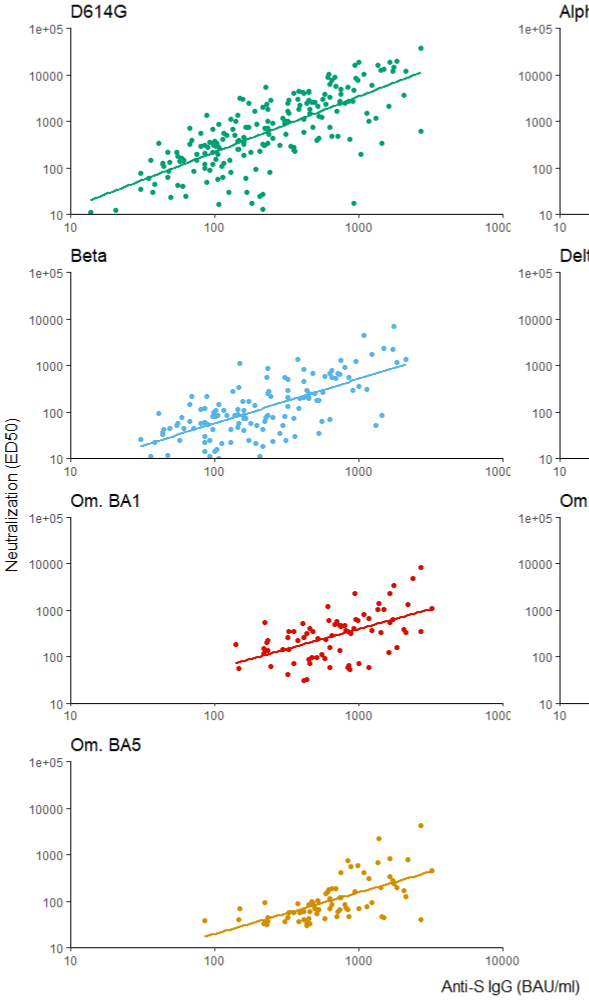

Figure 1: Linear regressions $ED_{50}^{\nu} = \beta^{\nu} Ab$ for each VoC from simultaneously measured neutralizing activity and binding antibody concentration. The censored data have been removed.
